# Pyogenic Liver Abscess, Bacteremia, and Meningitis with Hypermucoviscous *Klebsiella pneumoniae*: An Unusual Case Report in a Human T-Cell Lymphotropic Virus Positive Patient of Caribbean Origin in the United States

**DOI:** 10.1155/2013/676340

**Published:** 2013-12-30

**Authors:** Gargi Patel, Neha Shah, Roopali Sharma

**Affiliations:** ^1^SUNY Downstate Medical Center, Brooklyn, NY 11203, USA; ^2^Rockford Infectious Disease Consultants, Chicago, IL 61108, USA; ^3^Arnold & Marie Schwartz College of Pharmacy, SUNY College of Medicine, The Health Science Center at Brooklyn, Brooklyn, NY 11203, USA

## Abstract

Pyogenic liver abscess (PLA) is a potentially fatal disease. *Klebsiella pneumoniae* (*K. pneumoniae*) has replaced *Escherichia coli* (*E. coli*) as the predominant causative organism for pyogenic liver abscess. Over the years a unique form of community-acquired invasive *K. pneumoniae* infection of the liver has been well described in Southeast Asia. This has recently been linked to a virulent hypermucoviscous *K. pneumoniae* phenotype and to a specific genotype, *rmpA* positive. To our knowledge, we report the first case of PLA with bacteremia and meningitis in a Guyanese patient with the presence of *rmpA*-positive *K. pneumoniae* with laboratory evidence in North America.

## 1. Introduction

For the past few decades, an increased incidence of distinct community-acquired invasive *K. pneumoniae* syndrome in Taiwan and other Southeast Asian countries has been characterized by bacteremia, liver abscesses, and metastatic infections of the CNS, eye, and other sites [1, 2]. This invasive *K. pneumoniae* syndrome has also been reported as an emerging infection in the United States and other countries [3, 4]. Patients with this syndrome have been reported to be immunocompetent and with no underlying gastrointestinal pathology. Although diabetes mellitus seems to be an important risk factor in patients who acquire this infection, the mortality rate has been reported to be 2.8%–10.8% [5]. The invasiveness of *K. pneumoniae* is related to the hypermucoviscosity phenotype of the strain expressed by mucoviscosity-associated gene A (*magA*) and regulator of mucoid phenotype A (*rmpA*) [6, 7] The *rmpA* gene is a plasmid-mediated regulator of the extracapsular polysaccharide synthesis which is associated with hypermucoviscosity phenotype as well as with the *K. pneumoniae* invasive clinical syndrome [7]. We describe a case of pyogenic liver abscess, bacteremia, and meningitis with a hypermucoviscous *K. pneumonia rmpA* positive strain in a patient who was Human T-cell lymphotropic virus (HTLV-1) positive.

## 2. Case Report

A 68-year-old Guyanese woman with a history of cervical cancer (treated 20 years ago), Alzheimer's dementia, and depression presented to our institution in November 2011 with approximately a 2-day history of left shoulder pain radiating to the entire left side, headache, one episode of diarrhea, chronic abdominal pain, dizziness, blurriness, nuchal rigidity, photophobia, loss of appetite, and altered mental status. At admission, she was febrile (100.8°F) and tachycardic (99 beats per minute). The patient denied any sick contacts and had last traveled to Guyana two years ago. Of note, two months prior to this admission, she had presented to the emergency room with abdominal pain and was diagnosed with colonic diverticulosis without diverticulitis as evidenced by CT scan of abdomen which showed no other intraabdominal pathology. During this admission she received supportive care and was not placed on antibiotics.

**Figure 1:**
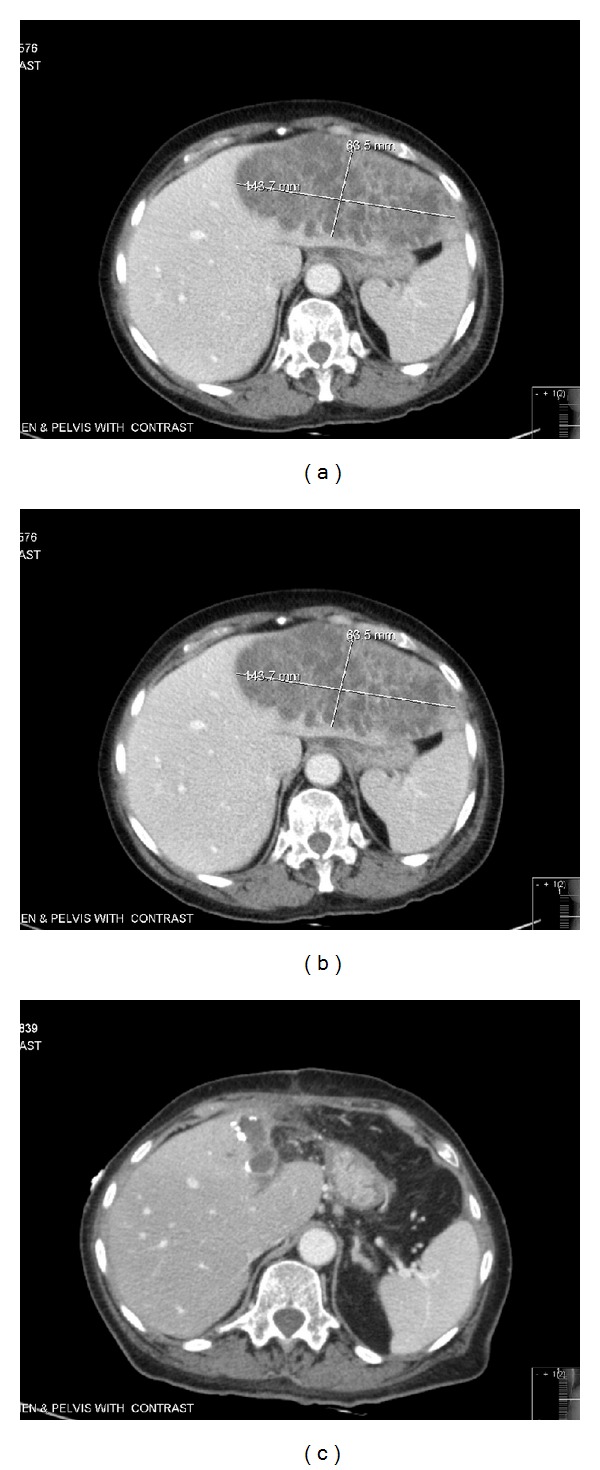
(a) Computed tomographic scan of the abdomen pelvis prior to IR drainage showing left liver abscess measuring 13.5 × 7.4 × 10.3 cm. (b) Computed tomographic scan of the abdomen pelvis 2 days after the IR drainage of 200 cc fluid, showing increase in abscess size to 16 × 7 cm. (c) Computed tomographic scan of the abdomen pelvis 5 weeks following left partial liver lobectomy and antibiotics showing abscess size reduced to 5.3 × 3.9 × 7 cm.

Patient underwent a lumbar puncture to rule out meningitis and blood cultures also were obtained. Lumbar puncture was consistent with bacterial meningitis (WBC 13920 cells/mm^3^, bands 30%, Segs 61%, serum protein 5.9, serum glucose 108). Lumbar puncture showed protein of 389 and glucose of 28, with WBC 950, RBC 1792, Segs 68%, and bands 28%. Gram stain from blood culture was reported as *Klebsiella Pneumoniae* which eventually was speciated as *Klebsiella Pneumoniae*. Urine cultures also revealed a gram-negative rod. CT scan of abdomen was obtained and revealed a multiloculated pyogenic liver abscess (13 × 7 × 10 cm) (Figure 1(a)). The patient underwent interventional radiology guided percutaneous drainage of the liver abscess with 2 pigtail catheter placement which drained minimally. All the cultures from cerebrospinal fluid, urine, blood, and liver abscess revealed the same pan-susceptible *Klebsiella pneumoniae*. She was started on meropenem 2 grams IV every eight hours.

Over the next two days her mental status further deteriorated, and she developed severe abdominal tenderness and worsening leukocytosis with bandemia. A repeat CT scan of abdomen/pelvis revealed that the liver abscess size had increased (16 × 7 cm) (Figure 1(b)). At this time she underwent open surgical drainage of the liver abscess with left partial liver lobectomy. Postoperatively, her hospital course was complicated with requirement of intubation, pressors, and prolonged ICU stay. She eventually stabilized clinically and was extubated successfully. The patient's antibiotic regimen included meropenem 2 grams IV every eight hours for a total of 7 days and then it was deescalated to ceftriaxone 2 grams IV every 12 hours for 2 weeks, followed by oral amoxicillin/clavulanate 875 mg twice a day for 3 weeks to finish a total of 6 weeks of antibiotic treatment.

**Figure 2:**
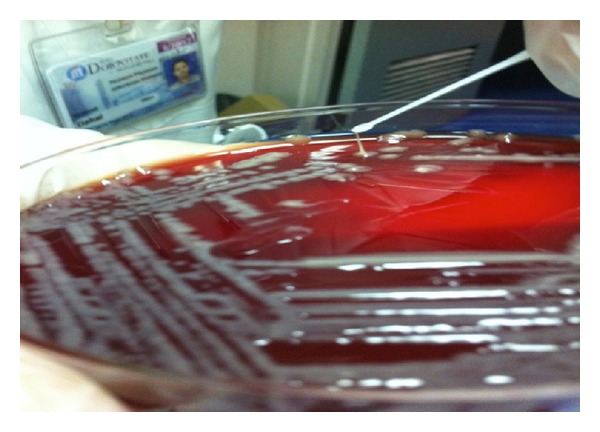
Image of positive string test result caused by *rmpA K. Pneumoniae*.

Genetic testing of the *K*. *pneumoniae* isolates was performed and revealed the presence of *rmpA* using the primers Forward (5′-ACTGGGCTACCTCTGCTTCA-3′) and Reverse (5′-CTTGCATGAGCCATCTTTCA-3′) [8]. Additionally, the patient's bacterial isolate from blood had a positive string test result, indicating hypermucoviscosity phenotype (Figure 2).

**Figure 3:**
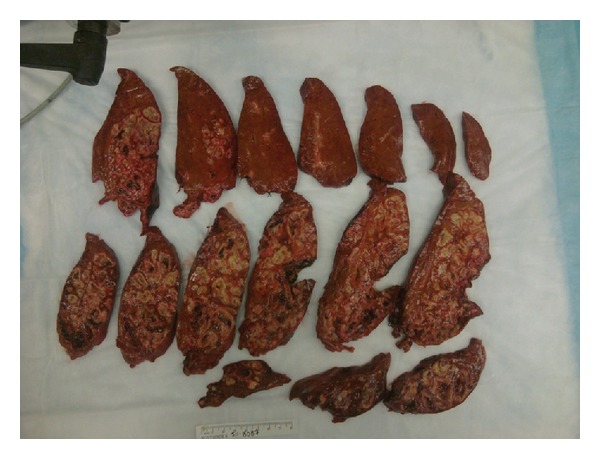
A cut surface of the liver with tan white nodules of varying size. The lesions are characterized by liquefactive necrosis with degenerating and necrotic liver cells.

Our patient had a prolonged hospital course, with complications including herpes simplex virus −2 (genital herpes) infection which was treated with acyclovir 400 mg three times daily for 7 days and diarrhea treated with metronidazole 500 mg by mouth three times daily for 14 days. Clostridium toxin assay was negative on three occasions. Workup for her diarrhea also included strongyloides stool serology (negative), HIV test (negative), and HTLV-1 serology (reactive, positive antibodies) which correlates to her Caribbean origin, where it is endemic. Although a complete ophthalmic examination was not performed during her hospitalization, she was clinically monitored and did not develop any symptoms of endophthalmitis. She completed a total of 6 weeks of antibiotics therapy and slowly recovered from this invasive *K*. *pneumoniae* liver abscess syndrome. A repeat CT of abdomen/pelvis was obtained one month after her partial liver lobectomy (Figure 3), and it revealed that the liver abscess had reduced in size (Figure 1(c)).

## 3. Discussion

Invasive syndromes of *K. pneumoniae* liver abscess with bacteremia and other metastatic infections including endophthalmitis in mainly poorly controlled diabetic patients was first reported in Taiwan in the mid-1980s [9]. During the 1990s and early 2000s, >900 patients with liver abscess due to *K. pneumoniae* were reported from East and Southeast Asian countries, whereas only ~50 cases were reported from countries outside of East and Southeast Asia in the same time period [10]. In 2005 and 2007, the first case reports that appeared from North America of *K. pneumoniae* bacteremia and liver abscess that confirmed the hypermucoviscous phenotype along with the *magA* and *rmpA* genotype, both patients were Asian and presented in diabetic ketoacidosis [11, 12]. Mortality rates have decreased substantially over the past several decades, with recent studies reporting rates of 11%–31% [1]. In mid-2000s a novel virulence gene *rmpA* and *magA* in *K. pneumoniae* strains was identified as a cause of this invasive *K. pneumoniae* syndrome and hypermucoviscosity; an extremely sticky phenotype of *K. pneumoniae* was associated with this pyogenic liver abscess. These hypermucoviscous *K. pneumoniae *abscesses are mainly community acquired and the strain positive for *rmpA* is significantly associated with the virulent hypermucoviscosity phenotype and purulent tissue infections in the liver and other organs [13]. This phenotype is confirmed by a positive string test result, in which a colony is lifted with a loop off the growing medium. A string greater than 5 mm is considered a positive result [14]. The average age of patients with *K. pneumoniae* liver abscess is 55–60 yr and is twice common in men than women [5]. Clinical symptoms such as fatigue, anorexia, and fever are some of the vague symptoms, and nausea/emesis, diffuse abdominal discomfort, pleuritic chest pain, right upper quadrant pain, and jaundice are more specific clinical clues [5]. The most common laboratory finding is leukocytosis (70–82%); alkaline phosphatase of 2-3 times the upper limit of normal is also noted [5]. These strains have been found to be susceptible to most antibiotics, including third and fourth generation cephalosporins, monobactam, carbapenems, and ciprofloxacin, whereas uniformly resistant to ampicillin [4, 10, 15, 16]. Host risk factors such as diabetes mellitus and Asian ancestry have been strongly associated with *K. pneumoniae* liver abscess and with development of metastatic complications [4].

This rare case of pyogenic liver abscess with hypermucoviscosity phenotype *K. pneumoniae* (*rmpA*) with metastatic complications of meningitis along with bacteremia and urinary tract infection were found in a Guyanese woman. This patient lacked traditional risk factors for patients who usually acquire this strain (i.e., she is not of Asian descent and not diabetic). We are unsure about an underlying link between her underlying HTLV-1 infection and her immune reaction to the *rmpA* strain of *K. pneumoniae*. Literature review suggests that this is a virulent strain regardless of immunocompetency. This strain is starting to spread into other demographics. The clinical significance of this strain is that infections are much more severe causing disseminated infection, rapidly developing (our patient had a normal CT scan 2 months before presentation), and more complicated (multiloculated liver abscess) which might require more invasive therapy such as open surgical drainage of the abscess and lobectomy of the liver for cure along with prolonged antibiotics for 6 weeks.
